# Climate Variability and Hemorrhagic Fever with Renal Syndrome Transmission in Northeastern China

**DOI:** 10.1289/ehp.0901504

**Published:** 2010-02-08

**Authors:** Wen-Yi Zhang, Wei-Dong Guo, Li-Qun Fang, Chang-Ping Li, Peng Bi, Gregory E. Glass, Jia-Fu Jiang, Shan-Hua Sun, Quan Qian, Wei Liu, Lei Yan, Hong Yang, Shi-Lu Tong, Wu-Chun Cao

**Affiliations:** 1 State Key Laboratory of Pathogen and Biosecurity, Beijing Institute of Microbiology and Epidemiology, Beijing, China; 2 Inner Mongolia Center for Disease Control and Prevention, Hohhot, China; 3 Department of Statistics, School of Public Health, Tianjin Medical University, Tianjin, China; 4 Discipline of Public Health, University of Adelaide, Adelaide, Australia; 5 Johns Hopkins Bloomberg School of Public Health, Baltimore, Maryland, USA; 6 Institute for Viral Disease Control and Prevention, Chinese Center for Disease Control and Prevention, Beijing, China; 7 School of Public Health, Queensland University of Technology, Queensland, Australia

**Keywords:** China, cross-correlation, forecast, hemorrhagic fever with renal syndrome, risk factors, time-series Poisson regression

## Abstract

**Background:**

The transmission of hemorrhagic fever with renal syndrome (HFRS) is influenced by climatic variables. However, few studies have examined the quantitative relationship between climate variation and HFRS transmission.

**Objective:**

We examined the potential impact of climate variability on HFRS transmission and developed climate-based forecasting models for HFRS in northeastern China.

**Methods:**

We obtained data on monthly counts of reported HFRS cases in Elunchun and Molidawahaner counties for 1997–2007 from the Inner Mongolia Center for Disease Control and Prevention and climate data from the Chinese Bureau of Meteorology. Cross-correlations assessed crude associations between climate variables, including rainfall, land surface temperature (LST), relative humidity (RH), and the multivariate El Niño Southern Oscillation (ENSO) index (MEI) and monthly HFRS cases over a range of lags. We used time-series Poisson regression models to examine the independent contribution of climatic variables to HFRS transmission.

**Results:**

Cross-correlation analyses showed that rainfall, LST, RH, and MEI were significantly associated with monthly HFRS cases with lags of 3–5 months in both study areas. The results of Poisson regression indicated that after controlling for the autocorrelation, seasonality, and long-term trend, rainfall, LST, RH, and MEI with lags of 3–5 months were associated with HFRS in both study areas. The final model had good accuracy in forecasting the occurrence of HFRS.

**Conclusions:**

Climate variability plays a significant role in HFRS transmission in northeastern China. The model developed in this study has implications for HFRS control and prevention.

Hantaviruses (family Bunyaviridae) are the causative agents of hemorrhagic fever with renal syndrome (HFRS) in Eurasia and hantavirus pulmonary syndrome (HPS) in the Americas (Peters et al. 1999; Schwarz et al. 2009). HFRS and HPS became a significant global health concern in the 1990s (Schmaljohn and Hjelle 1997). HFRS is epidemic in China, where it accounts for 90% of the total reported cases in the world (Yan et al. 2007).

The typical clinical symptoms include fever, hemorrhage, headache, back pain, abdominal pain, acute renal dysfunction, and hypotension (Ministry of Health 1998). In China, HFRS is predominantly caused by *Hantaan* virus and *Seoul* virus, which have *Apodemus agrarius* and *Rattus norvegicus*, respectively, as their rodent hosts (Yan et al. 2007). Transmission of hantaviruses from rodents to humans is believed to occur through inhalation of aerosols contaminated by virus shed in excreta, saliva, and urine of infected animals (Clement 2003; Shi 2003; Song 1999).

The number of HFRS cases varies geographically, seasonally, and interannually in China (Fang et al. 2006) and is influenced by the density and hantavirus infection rate of host rodents, as well as the contact rate between rodents and human beings (Jiang et al. 2006; Zhang et al. 2009). Infection rates and population dynamics of hosts are thought to be influenced by climatic factors (Bi et al. 1998, 2002; Engelthaler et al. 1999; Ernest et al. 2000; Glass et al. 2007; Langlois et al. 2001; Madsen and Shine 1999; Yan et al. 2007; Zhang et al. 2009). For example, increased production of grass seed after heavy precipitation, as a result of the El Niño Southern Oscillation (ENSO), has been linked to higher *Peromyscus maniculatus* rodent densities—the reservoirs of Sin Nombre virus—in North America (Engelthaler et al. 1999; Glass et al. 2002; Hjelle and Glass 2000; Tamerius et al. 2007). Similarly, a positive relationship has been reported for tree seed production, milder climate, and hantavirus disease incidence in Europe (Clement et al. 2009; Tersago et al. 2009). These climatic conditions have been proposed as early warning indicators of potential outbreaks of HFRS (Pettersson et al. 2008; Schwarz et al. 2009).

Previous studies in China also demonstrated that the transmission of HFRS was influenced by such environmental elements as elevation, precipitation, temperature, vegetation type, land use, and ENSO (Bi et al. 1998; 2002; Yan et al. 2007; Zhang et al. 2009). These studies have suggested that the climatic variables related to rodent population dynamics might serve as indicators for the risk of HFRS transmission. However, the quantitative relationship between climate variation and the transmission of HFRS remains to be determined, especially in northeastern China, where its incidence is higher than in other parts of China and where different weather and geographic characteristics prevail.

In this study, we examined the potential impact of climate variability on the transmission of HFRS and developed a climate-based forecasting model for the control and prevention of HFRS.

## Materials and Methods

### Study areas

**Figure 1:**
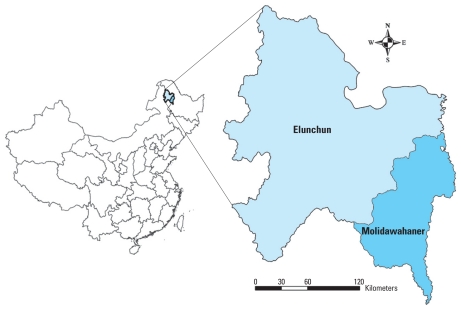
Study areas in China.

Northeastern China has the highest HFRS incidence in the country where the illness has reached epidemic levels (Fang et al. 2006; Yan et al. 2007). From 1997 to 2007, Elunchun County (population size, 292,097) and Molidawahaner County (population size, 294,501), located in the east of the Da-Xing-An Mountains (Figure 1), reported the highest incidences of HFRS in China where the annual average incidence reached 59.69 and 61.12 per 100,000, respectively. Both are minority (non-Han Chinese) counties with tourist attractions, and endemic HFRS has had a considerable impact on tourism and economic development. Elunchun county has a frigid–temperate climate with windy, dry springs; rainy, sunny, short summers; and long, cold winters. Rain fall occurs mostly during the summer months between June and August. Almost half (48.9%) of the county is covered with forests. Molidawahaner county has a moderate–temperate climate with clearly delineated seasonal change. The annual rainfall is about 500 mm, with most of the rain fall in the summer months. Forestry and soybean agriculture are the main occupational activities in both counties.

### Data collection

Data on the notified monthly HFRS cases in Elunchun and Molidawahaner from 1997 to 2007 were provided by the Center for Disease Control and Prevention of the Inner Mongolia Autonomous Region. In this study, all the HFRS cases were confirmed according to the diagnostic from the Ministry of Health of the People’s Republic of China (Ministry of Health 1998). A confirmed case of HFRS was defined as a person who had traveled to the HFRS endemic area or who had come into contact with rodent feces, saliva, and urine within 2 months before the onset of illness, and who had an acute illness characterized by abrupt onset of at least two of the following clinical features: fever, chills, hemorrhage, headache, back pain, abdominal pain, acute renal dysfunction, and hypotension. In addition, the person had to have had at least one of the laboratory criteria for diagnosis: a positive result for hantavirus-specific immunoglobulin M, or a 4-fold rise in titers of hantavirus-specific immunoglobulin G, or a positive result for hantavirus-specific ribonucleic acid by reverse transcription polymerase chain reaction in clinical specimens, or hantavirus isolated from clinical specimens.

Local climate data on monthly rainfall, relative humidity (RH), and land surface temperature (LST) for the study period were obtained from the Chinese Bureau of Meteorology (China Meteorological Data Sharing Service System 2004). ENSO is the most important coupled ocean–atmosphere phenomenon that affects global climate variability and the climate in China (Huang and Wu 1989). The multivariate ENSO index (MEI) was used as an indicator of the global climate pattern available from the Earth System Research Laboratory of the National Oceanic and Atmospheric Administration (2009).

### Statistical analysis

We summarized a description of climate variables and disease incidence and performed cross-correlation analysis to assess the associations between climate variables and the number of HFRS cases for a range of lags (Brockwell and Davis 2002). In this study, we included lags of up to 6 months and presented the climatic variables with the maximum correlation coefficients. Time-series Poisson regression analysis was used to examine the independent contribution of climatic variables to HFRS transmission. Time series analysis has been used extensively to study the association between climate variability and disease transmission (Bi et al. 2008; Constantin de Magny et al. 2008; Hashizume et al. 2009; Naish et al. 2006; Tong and Hu 2001; Zhang et al. 2007; Zhou et al. 2004).

We performed time-series Poisson regression analysis that allowed for autocorrelation, seasonality, and lag effects after correcting for overdispersion (Bi et al. 2008). Temporal associations between climate variability and the disease are often confounded by patterns in seasonal and long-term trends (i.e., interannual change trend) (Hashizume et al. 2009). To control the impact of seasonality and long-term trends, we created indicator variables for “month” and “year” of onset in the model (Bi et al. 2008; Hashizume et al. 2009). Climatic variables for the months preceding the HFRS outbreaks have been shown to be important (Glass et al. 2002, 2007; Hjelle and Glass 2000). Thus, to account for the lagged effect of the climatic variables on the number of HFRS cases, we incorporated climatic variables over a range of lags into the model (Brockwell and Davis 2002; Ostrom 1978). We used the basic Poisson regression model for this study:


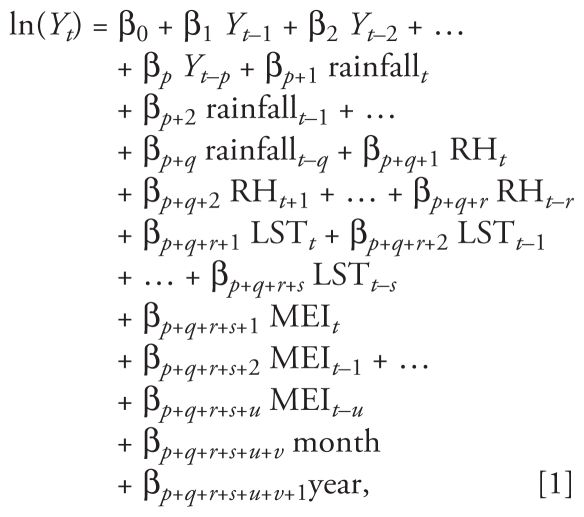


where month as the dummy variable and the others as continuous variables were included in the model, and *p*, *q*, *r*, *s*, *t*, *u*, and *v* were lags determined by correlation analyses (Bi et al. 2008); β denotes the regression coefficients, and *Y* represents the number of cases. We used a stepwise approach in the analysis to retain variables that contributed to a significant improvement in model fit as determined by the maximum likelihood (α = 0.05) (Dupont 2002). Associations between determinants and notifications of HFRS cases are presented as incidence rate ratios (IRRs) that were derived from estimated regression parameters from the final model. All estimates of IRR were complemented by a 95% confidence interval (CI) and *p*-value. We determined the goodness-of-fit of the models using both time series (e.g., autocorrelation function and partial autocorrelation function of residuals) and the pseudo-*R*^2^ (Tabachnick and Fidell 2001). Finally, the results from the empirical data during the period of January 1997 to December 2005 were used to develop the models, and data from January 2006 to December 2007 were used to validate the forecasting ability of the models. We used SPSS software (version 16.0; SPSS Inc., Chicago, IL, USA) to perform all the analyses.

## Results

**Figure 2:**
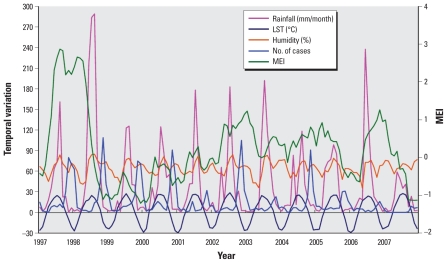
Temporal variation in climatic variables and the number of HFRS cases in Elunchun, 1997–2007.

Elunchun had 1,918 notified HFRS cases during the study period, with an annual average incidence of 59.69 per 100,000. We observed a peak of HFRS cases in winter (November through January). The monthly mean LST was 1.7 °C, and the monthly mean rainfall was 40.5 mm during the study period. Figure 2 shows the temporal variation in climatic variables and the number of cases during the study period.

**Figure 3:**
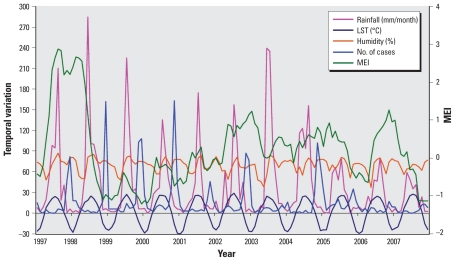
Temporal variation in climatic variables and the number of HFRS cases in Molidawahaner, 1997–2007.

A total of 1,980 HFRS cases were reported in Molidawahaner during the study period, with an annual average incidence of 61.12 per 100,000. We also observed a peak of HFRS cases in winter. The monthly mean for LST and for rainfall were 1.3 °C and 39.4 mm, respectively. Figure 3 shows the temporal variation in climatic variables and the number of cases.

In both study locations, rainfall, LST, RH, and MEI were significantly correlated with the monthly reports of HFRS with lags of 3–5 months (Table 1). However, the lag effects of all these climatic variables, except MEI, differed somewhat between the two study areas.

For Elunchun, the Poisson regression model showed that the occurrence of HFRS cases was first-order autoregressive. This findng indicated that the number of notified cases in the current month was related to the number of cases that occurred in the previous month (Table 2). Seasonality and long-term trends were also significantly associated with HFRS notifications. More important, after controlling for the autocorrelation, seasonality, and long-term trend, rainfall at a lag of 3 months, LST at a lag of 4 months, RH at a lag of 3 months, and MEI at a lag of 4 months appeared to play significant roles in the transmission of HFRS. The final Poisson regression model suggests that a 1°C increase in the monthly mean LST may be associated with an 11.4% (95% CI, 1.8–21.9%) increase in HFRS cases. A 1-mm/day increase in monthly mean rainfall, 1% RH rise, and 1-unit MEI rise were associated with 1.1% (95% CI, 0.2–1.9%), 2.9% (95% CI, 1.4–4.4%), and 55.3% (95% CI, 20.1–89.6%) increases in HFRS cases, respectively.

**Figure 4:**
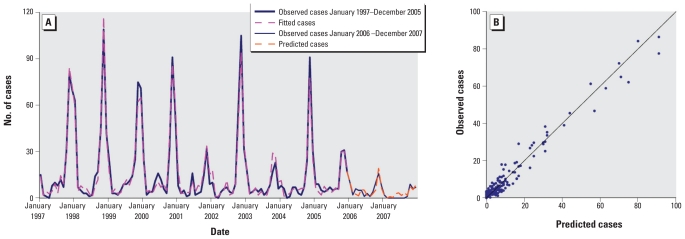
Observed versus predicted HFRS cases in Elunchun: temporal dynamics (*A*) and scatterplot (*B*).

**Figure 5:**
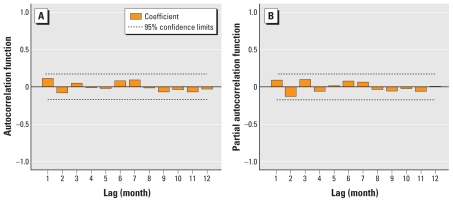
Autocorrelation (*A*) and partial autocorrelation (*B*) of residuals in Elunchun

The observed and expected number of cases from the final model matched reasonably well for Elunchun, as did the 2-year forecast (Figure 4A). Figure 4B compares observed numbers of HFRS cases with predicted numbers from the fitted model; the pseudo-*R*^2^ value for the fitted model was 79.43%. The goodness-of-fit analyses showed no significant autocorrelation between residuals at different lags in the final model (Figure 5).

For Molidawahaner, the time-series Poisson regression model (Table 3) was also first-order autoregressive. After controlling for the autocorrelation, seasonality, and long-term trend, rainfall at a lag of 4 months, LST at a lag of 5 months, RH at a lag of 4 months, and MEI at a lag of 4 months were significantly associated with HFRS. The final model indicated that a 1°C increase in the monthly mean LST was associated with a 16.8% (95% CI, 11.5–22.3%) increase in HFRS cases. A 1-mm/day increase in monthly mean rainfall, 1% RH rise, and 1-unit MEI rise were associated with 0.5% (95% CI, 0.2–0.8%), 3.2% (95% CI, 1.7–4.6%), and 73.6% (95% CI, 41.8–95.6%) increases in HFRS cases, respectively.

**Figure 6:**
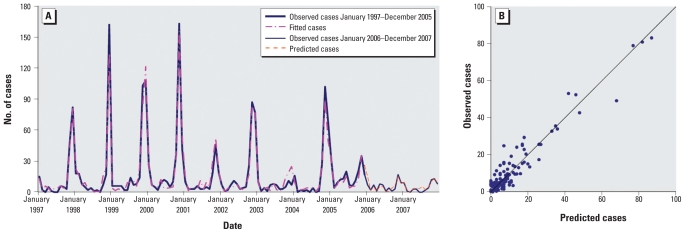
Observed versus predicted HFRS cases in Molidawahaner: temporal dynamics (*A*) and scatterplot (*B*).

The results of the model validation also were similar in Molidawahaner (Figure 6); the pseudo-*R*^2^ value for the fitted model was 75.91%. We found no significant autocorrelation between residuals at different lags in the final model (data not shown).

## Discussion

A key finding from this study was that climate variability is an important predictor of the intensity of HFRS transmission in northeastern China. We found a consistent relationship between climatic factors with lags of 3–5 months and HFRS transmission. This lead time is of practical importance in predicting epidemics of HFRS and giving health authorities sufficient time to formulate plans, disseminate warnings, and implement public health interventions, such as vaccinating high-risk populations, killing the rodent hosts, and managing environments for the prevention and control of the disease (Ministry of Health 1998). Interestingly, despite the geographical proximity of Elunchun and Molidawahaner, lag periods for associations of all these climatic variables, except MEI, with HFRS cases were somewhat different, such that climate variables in Elunchun typically appeared to influence HFRS case reports 1 month earlier than they did in Molidawahaner. It is unclear if this difference is due to a systematic difference in reporting time, although this seems unlikely during this long time period, or reflects differences in the biological characteristics of viral transmission.

Molidawahaner is somewhat cooler and drier than Elunchun, and this may affect the dynamics of reservoir populations as well as viral transmission within reservoir populations. These regional differences suggest that variation of other local environmental variables such as types of land use and patterns of agricultural production may also affect the short-term dynamics of HFRS epidemics (Bi et al. 2002; Yan et al. 2007; Zhang et al. 2009).

A deeper understanding of the relationship between climate variability and disease dynamics provides a foundation for anticipating the health effects of global climate change (Brownstein et al. 2003; Patz et al. 2002). In this study, HFRS was consistently and strongly associated with temperature, precipitation, RH, and ENSO index in northeastern China. Similarly, associations with climate variability have been found for other vector-borne diseases and zoonoses, including malaria (Thomson et al. 2005; Zhou et al. 2004), dengue fever (Cazelles et al. 2005; Hales et al. 2002), Ross River virus infection (Tong and Hu 2001, 2002), cutaneous leishmaniasis (Chaves and Pascual 2006; Franke et al. 2002), and plague (Nakazawa et al. 2007; Stenseth et al. 2006).

Temperature affects rodent dynamics and activity as well as infectivity of hantavirus (Aars and Ims 2002; Bi et al. 2002; Hardestam et al. 2007; Jensen 1982; Pucek et al. 1993; Zheng et al. 2008). We used LST, rather than air temperature, in the present study because it more directly influences the abundance and distribution of rodents (Yan et al. 2007; Zheng et al. 2008). In this study, changes in LST appeared to have a greater impact on the persistence and transmission of HFRS than did rainfall. In cooler climates, warmer temperatures may allow reservoirs to survive more easily in winters that normally would have limited their populations and to cause rodents to reach maturity much faster than lower temperatures (Aars and Ims 2002; Clement et al. 2009; Rezende et al. 2003; Tersago et al. 2009). Transmission and persistence of hantavirus may therefore be enhanced under warmer conditions because more rodents become infectious within their life span.

Variability in rainfall can have important consequences for the transmission of rodent-borne diseases (Bi et al. 2002; Engelthaler et al. 1999; Ernest et al. 2000; Hjelle and Glass 2000; Madsen and Shine 1999; Nakazawa et al. 2007; Stenseth et al. 2006; Yan et al. 2007) by increasing the growth of vegetation that either directly or indirectly serves as food for rodent hosts, resulting in larger rodent populations. Increased rodent densities can be associated with increased viral transmission among rodent populations and larger numbers of dispersing animals, which in turn lead to increased transmission to humans who contact these animals (Engelthaler et al. 1999; Glass et al. 2000, 2007; Yates 2002). About 90% of the HFRS epidemic foci in China are in low-lying regions with moist or semimoist soils (Bi et al. 2002; Yan et al. 2007). This study corroborates previous findings that rainfall is an important factor in the transmission of HFRS in China.

RH was also positively associated with the transmission of HFRS in northeastern China. Higher levels of humidity may be indicative of moisture influencing the survival of rodent hosts but also is known to influence the infectivity and stability of the virus in the *ex vivo* environment (Hardestam et al. 2007; Vickery and Bider 1981; Zheng et al. 2008). Previously, we reported a close association between the distribution of hantavirus-infected *R. norvegicus* and wet habitats (Jiang et al. 2006; Zhang et al. 2009). The prevalence of infection among rodents trapped on north-facing slopes was higher than that among rodents trapped on south-facing slopes. This finding may reflect the higher humidity on the north-facing slopes.

MEI, as an indicator of global climate pattern, was an important contributor for characterizing changing patterns of HFRS, which supports the notion that large-scale climate indices may be critical for forecasting patterns of disease risk, especially among larger geographic areas (Bi and Parton 2003; Hallett et al. 2004; Stenseth et al. 2003). The MEI is favored over other conventional indices because it integrates more information, reflects the nature of the coupled ocean–atmosphere system better than either component alone, and is less vulnerable to non-ENSO-related variability and to occasional data glitches in the monthly cycles (Chaves and Pascual 2006; Wolter and Timlin 1998). It also has the practical utility in analyses by reducing the number of predictors, thus avoiding problems of collinearity in the predictor matrix (Chaves and Pascual 2006; Stenseth et al. 2003).

Climate variability can affect the dynamics of infectious diseases through either linear or nonlinear pathways. It can affect several biological traits of the rodent hosts, from individual life histories to population dynamics (Ernest et al. 2000; Hallett et al. 2004; Madsen and Shine 1999), and modify several factors that determine the context of disease transmission, both of which are known as important risk factors for rodent-borne diseases (Bi et al. 1998, 2002). The local climate is more likely to directly affect the life cycle dynamics (e.g., reproductive rates, incubation periods) of the disease agents themselves, whereas larger scale factors could also influence broader ecological processes that have important and possibly nonlinear impacts on the disease transmission dynamics (Hallett et al. 2004; Zheng et al. 2008).

This is the first study to look for the responses of HFRS to climate variability at a fine timescale in two locations where hundreds of thousands of people are at risk. The statistical approaches using various sources of data identified models that accounted for a high proportion of the variation in HFRS case numbers and forecast HFRS with good accuracy.

The limitations of this study should also be acknowledged. First, the data are from a passive surveillance system, so the quality of data is not as good as that collected from active surveillance. Some cases of HFRS might go unreported because of their milder clinical symptoms. It is likely that underreported cases influence the precision of models. However, it seems unlikely that disease severity would have an interannual component that would influence the general pattern of the observed results. Second, not all of the variation in the occurrence of HFRS cases in each region is caused by climate alone. In addition to factors such as virus and host dynamics that may be influenced directly by climate, human activities and movement, socioeconomic status, and population immunity may contribute to the transmission of HFRS. Clearing forests for agricultural use and urbanization may increase the potential for HFRS transmission (Zhang et al. 2009). Tourism and travel have also become important mechanisms for facilitating the transmission of HFRS. However, data were unavailable on many of these factors. Third, the “validation” of the final model is still relatively short-term and may not remain as predictive over time because of unincorporated covariates or ecological changes associated with natural and human factors that alter the relationships.

## Conclusion

The results of this study suggest that antecedent patterns of LST, ENSO index, precipitation, and RH were among the key determinants of HFRS transmission in northeastern China that are easily monitored over large geographic areas. As such, whether directly influencing virus transmission or acting as surrogates for those factors, they act as a basis for a forecasting system to control and prevent rodent-borne diseases. Early warning systems based on climatic forecasting can assist in improving reservoir control and personal protection. The results of this study provide an impetus to build a predictive capacity for HFRS epidemics and to develop an early warning system for enhancing public health measures, especially for developing countries and areas of the world that are vulnerable to the impact of climate change.

## Figures and Tables

**Table 1:** Maximum cross-correlation coefficients of monthly climatic variables and notifications of HFRS: Elunchun and Molidawahaner, China, 1997–2007.

	Elunchun			Molidawahaner		
Climate variable	Maximum coefficient^a^	Lag values (month)	*p*-Value	Maximum coefficient^a^	Lag values (month)	*p*-Value
Rainfall	0.53	3	0.000	0.46	4	0.000
LST	0.61	4	0.000	0.53	5	0.000
RH	0.27	3	0.015	0.38	4	0.000
MEI	0.24	4	0.042	0.25	4	0.008

a Chi-square test for cross-correlations.

**Table 2:** Parameters estimated by time-series Poisson regression for HFRS in Elunchun, 1997–2007.^a^

Variable	IRR	95% CI	*p*-Value^b^
No. of cases, 1-month lag	1.014	1.004–1.025	0.006
Mean rainfall (mm), 3-month lag	1.011	1.002–1.019	0.017
Mean LST (°C), 4-month lag	1.114	1.018–1.219	0.019
RH (%), 3-month lag	1.029	1.014–1.044	0.000
MEI, 4-month lag	1.553	1.201–1.896	0.001
Year of onset	0.992	0.984–0.999	0.032

IRR, incidence rate ratio.

a Dummy variables for month were included in the final model.

b Wald chi-square test.

**Table 3:** Parameters estimated by time-series Poisson regression for HFRS in Molidawahaner, 1997–2007.^a^

Variable	IRR	95% CI	*p*-Value^b^
No. of cases, 1-month lag	1.009	1.006–1.011	0.000
Mean rainfall (mm), 4-month lag	1.005	1.002–1.008	0.000
Mean LST (°C), 5-month lag	1.168	1.115–1.223	0.000
RH (%), 4-month lag	1.032	1.017–1.046	0.000
MEI, 4-month lag	1.736	1.418–1.956	0.005
Year of onset	0.978	0.964–0.992	0.002

a Dummy variables for month were included in the final model.

b Wald chi-square test.
